# Phenotypic Clustering of Patients With Newly Diagnosed Coronary Artery Disease Using Cardiovascular Magnetic Resonance and Coronary Computed Tomography Angiography

**DOI:** 10.3389/fcvm.2021.760120

**Published:** 2021-11-18

**Authors:** Théo Pezel, Thierry Unterseeh, Thomas Hovasse, Anouk Asselin, Thierry Lefèvre, Bernard Chevalier, Antoinette Neylon, Hakim Benamer, Stéphane Champagne, Francesca Sanguineti, Solenn Toupin, Philippe Garot, Jérôme Garot

**Affiliations:** ^1^Institut Cardiovasculaire Paris Sud, Cardiovascular Magnetic Resonance Laboratory, Hôpital Privé Jacques CARTIER, Ramsay Santé, Massy, France; ^2^Department of Cardiology, Lariboisiere Hospital – APHP, INSERM UMRS 942, University of Paris, Paris, France; ^3^Institut Cardiovasculaire Paris Sud, Department of Computed Tomography Imaging and Interventional Cardiology, Hôpital Privé Jacques CARTIER, Ramsay Santé, Massy, France; ^4^Independent Biostatistician, Paris, France; ^5^Scientific Partnerships Division, Siemens Healthcare France, Saint-Denis, France

**Keywords:** clustering, phenomapping, stress cardiovascular magnetic resonance imaging, coronary computed tomographic angiogram (CCTA), outcomes, ischemia, coronary artery disease

## Abstract

**Background:** Epidemiological characteristics and prognostic profiles of patients with newly diagnosed coronary artery disease (CAD) are heterogeneous. Therefore, providing individualized cardiovascular (CV) risk stratification and tailored prevention is crucial.

**Objective:** Phenotypic unsupervised clustering integrating clinical, coronary computed tomography angiography (CCTA), and cardiac magnetic resonance (CMR) data were used to unveil pathophysiological differences between subgroups of patients with newly diagnosed CAD.

**Materials and Methods:** Between 2008 and 2020, consecutive patients with newly diagnosed obstructive CAD on CCTA and further referred for vasodilator stress CMR were followed for the occurrence of major adverse cardiovascular events (MACE), defined by cardiovascular death or non-fatal myocardial infarction. For this exploratory work, a cluster analysis was performed on clinical, CCTA, and CMR variables, and associations between phenogroups and outcomes were assessed.

**Results:** Among 2,210 patients who underwent both CCTA and CMR, 2,015 (46% men, mean 70 ± 12 years) completed follow-up [median 6.8 (IQR 5.9–9.2) years], in which 277 experienced a MACE (13.7%). Three mutually exclusive and clinically distinct phenogroups (PG) were identified based upon unsupervised hierarchical clustering of principal components: (PG1) CAD in elderly patients with few traditional risk factors; (PG2) women with metabolic syndrome, calcified plaques on CCTA, and preserved left ventricular ejection fraction (LVEF); (PG3) younger men smokers with proximal non-calcified plaques on CCTA, myocardial scar, and reduced LVEF. Using survival analysis, the occurrence of MACE, cardiovascular mortality, and all-cause mortality (all *p* < 0.001) differed among the three PG, in which PG3 had the worse prognosis. In each PG, inducible ischemia was associated with MACE [PG1, Hazards Ratio (HR) = 3.09, 95% CI, 1.70–5.62; PG2, HR = 3.62, 95% CI, 2.31–5.7; PG3, HR = 3.55, 95% CI, 2.3–5.49; all *p* < 0.001]. The study presented some key limitations that may impact generalizability.

**Conclusions:** Cluster analysis of clinical, CCTA, and CMR variables identified three phenogroups of patients with newly diagnosed CAD that were associated with distinct clinical and prognostic profiles. Inducible ischemia assessed by stress CMR remained associated with the occurrence of MACE within each phenogroup. Whether automated unsupervised phenogrouping of CAD patients may improve clinical decision-making should be further explored in prospective studies.

## Introduction

Individualized cardiovascular risk stratification and tailored prevention are essential to limit the ever-increasing burden of coronary artery disease (1). However, diagnostic and preventive strategies based on the management of traditional risk factors may be limited. Beyond traditional risk factors, non-invasive imaging techniques may provide important data to improve risk stratification (2, 3). Coronary computed tomography angiography (CCTA) provides detailed information on CAD burden (4). Numerous studies have shown the independent prognostic value of CCTA above traditional risk factors (5–7). However, the epidemiological characteristics and prognostic profiles of patients with newly diagnosed CAD on CCTA are heterogeneous, particularly in terms of age, distribution of traditional risk factors, CAD burden, and left ventricular (LV) abnormalities.

Stress cardiovascular magnetic resonance (CMR) can assess the presence of both inducible ischemia and myocardial scar. In patients with known or suspected CAD, numerous large studies have shown the incremental prognostic value of inducible ischemia or unrecognized myocardial infarction (MI) by stress CMR, above traditional risk factors (8–11). Notably, perfusion stress CMR may improve diagnostic yield in patients with high coronary artery calcium (CAC) score (12), emphasizing the complementary roles of anatomical and functional data.

Whereas, traditional statistical analyses are built on a priori hypotheses, cluster analysis using unsupervised algorithms provides new perspectives for accurate phenotyping in heterogeneous populations (13). We hypothesized that a clustering approach could highlight different phenogroups with specific clinical and prognostic profiles in patients with newly diagnosed CAD. The study aimed to (i) identify robust phenogroups among patients with newly diagnosed CAD using an unsupervised clustering approach based on clinical, CCTA, and CMR data; (ii) describe the clinical profiles of the patients involved; (iii) compare outcomes in the different phenogroups; (iv) investigate the prognostic value of inducible ischemia on stress CMR in each phenogroup.

## Materials and Methods

### Study Population

**Figure 1:**
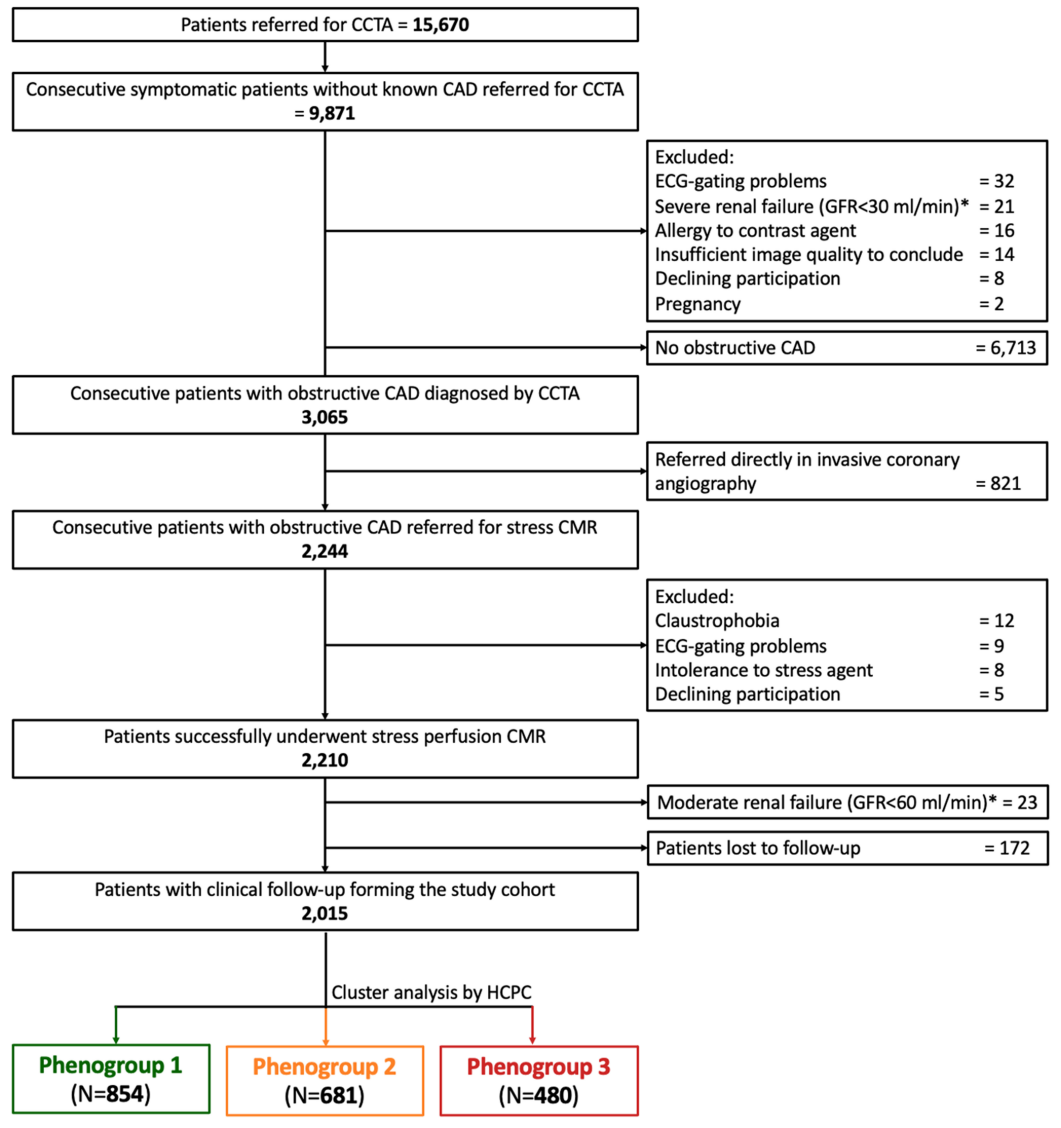
Flowchart of the study. CAD, coronary artery disease; CCTA, coronary computed tomography angiography; CMR, cardiac magnetic resonance; GFR, glomerular filtration rate; HCPC, hierarchical clustering on principal components.

Between December 2008 and January 2020, we conducted a single-center longitudinal study in an EACVI-accredited imaging laboratory, with a retrospective screening of all consecutive symptomatic patients with newly diagnosed obstructive CAD on CCTA, defined by the presence of at least one ≥ 50% stenosis (5, 14). Patients with previously known CAD before index CCTA were excluded. All patients with moderate renal failure defined by glomerular rate <60 ml/min/1.73 m^2^ were excluded to avoid any confounding factor in the analysis of coronary plaque composition. Those symptomatic patients with ≥1 coronary stenosis on CCTA and further referred for stress CMR (within 3 months after index CCTA) to evaluate the significance of that stenosis were included. The flowchart of the study is depicted in Figure 1. Symptomatic patients were defined by the presence of angina or dyspnea on exertion. In the first place, patients with a high-grade > 90% stenosis on CCTA were directly referred for invasive coronary angiography without stress CMR exam. The main exclusion criteria were known CAD, contraindication to CCTA, CMR, or dipyridamole (detailed list in Supplementary Material 1). Clinical data including symptoms were collected according to the medical history and clinical examination on the day of CCTA. All patients gave informed written consent for CCTA, CMR, and enrolment in the clinical research study. The study was approved by the local Ethics Committee of our Institutions and conducted in accordance with the Declaration of Helsinki. This study followed the STROBE reporting guidelines for cohort studies. Clinical, CCTA, and CMR data were prospectively recorded into a dedicated database (Clinigrid software, Hemolia, France).

### Patients Follow-Up and Clinical Outcome

The follow-up consisted of a clinical visit as part of usual care (67%) or by direct contact with the patient or the referring cardiologist (33%). Data collection was ended on January 2021. Cardiovascular (CV) events were checked by medical reports collected from the affiliated hospitals. The primary composite endpoint was the occurrence of at least one of the combined major adverse clinical events (MACE), defined as CV mortality or non-fatal MI. The secondary endpoints were CV mortality and all-cause mortality. All these clinical events were defined according to standardized definitions (15, 16), and are detailed in Supplementary Material 2. Three patients who experienced peri-procedural events after percutaneous coronary intervention (PCI) or coronary artery bypass grafting (CABG) <90 days after the index CCTA examination were excluded.

### CCTA Protocol and Analysis

Between December 2008 and January 2020, all CCTA studies were performed on multidetector CT scanners with ≥64 detector rows (Brilliance 64, Philips Healthcare, Eindhoven, Netherlands, between 2008 and 2011; Philips iCT 128, between 2012 and 2018; Aquilion One Genesis, Canon Medical Systems, Otawara, Japan from 2019 to 2020), and the imaging protocol adhered to the Society of CV Computed Tomography guidelines on appropriateness and performance of CCTA available at the time of scanning (4, 17–19). Each CCTA protocol is detailed in Supplementary Material 3 and radiation exposure assessment is described in Supplementary Material 4.

Coronary computed tomography angiography data were interpreted using multi-planar reconstruction and maximum intensity projections. The data were analyzed based on the 16-segment coronary artery model (20). Coronary segments were scored visually for the presence and composition of coronary plaque and degree of luminal stenosis. In each coronary segment, coronary atherosclerosis was defined as any tissue structure > 1 mm either within the coronary artery lumen or adjacent to the coronary artery lumen that could be discriminated from surrounding pericardial tissue, epicardial fat, and the vessel lumen itself, as previously described (21). In each coronary artery segment, plaques were classified as non-calcified, mixed, or calcified, as previously defined (5). Following the CAD-RADS classification (22), the severity of CAD was categorized according to the highest value of stenosis of the diameter among segments: normal (0% luminal stenosis), non-obstructive CAD (1–49% luminal stenosis), obstructive CAD (50–69% luminal stenosis), or severe obstructive CAD (≥70% luminal stenosis). Within the category of obstructive CAD, we further divided CCTA findings as 1-, 2-, or 3-vessel disease/left main according to the number of major epicardial vessels with the presence of ≥50% stenosis. The presence of ≥50% stenosis in the left main coronary artery was considered a 3-vessel disease equivalent. The number of segments with any plaque or stenosis, a specific plaque composition, specific luminal stenosis, or a specific topography of the plaque (proximal vs. no proximal) were assessed for each patient, following the same method analysis of previous studies (5, 14).

### Stress CMR Protocol

The detailed stress CMR protocol has been previously published (23, 24) and is described in Supplementary Material 5. Briefly, CMR was performed on a 1.5 T scanner (MAGNETOM Espree and Aera, Siemens Healthcare, Erlangen, Germany). Vasodilation was induced with dipyridamole injected at 0.84 mg/kg over 3 min. Then, a bolus of gadolinium-based contrast agent (Dotarem^®^, Guerbet, France, 0.1 mmol/kg) was injected at a rate of 5 ml/s. Stress perfusion imaging was performed using an ECG-triggered saturation-prepared balanced steady-state free-precession sequence (Siemens, Erlangen, Germany). A series of six slices (four short-axis views, a two-chamber, and a four-chamber view) were acquired every other heartbeat. Then, 10 min after contrast injection, a breath-hold contrast-enhanced 3D T1-weighted inversion-recovery gradient-echo sequence was acquired to detect late gadolinium enhancement (LGE). CMR sequence parameters are detailed in Supplementary Material 6.

### CMR Image Analysis

Left ventricular volumes and functions were quantified on the short-axis cine stack. Stress perfusion and LGE images were evaluated according to the 17-segment model of the American Heart Association (25). The analysis of perfusion images was done visually by two experienced physicians blinded to clinical and follow-up data. Inducible ischemia was defined as a subendocardial perfusion defect that (1) occurred in at least one myocardial segment, (2) persisted for at least three phases beyond peak contrast enhancement, (3) followed a coronary distribution, and (4) in the absence of co-location with LGE in the same segment (8). An unrecognized MI was defined by LGE with ischemic patterns defined by subendocardial or transmural LGE (26). LGE with non-ischemic patterns was defined by any location that did not involve the subendocardium and was not transmural. For LGE with ischemic patterns, a myocardial segment was considered viable if LGE thickness was <50% and non-viable when LGE thickness was ≥ 50% of the myocardial wall (27). The total number of ischemic and LGE segments was assessed for each patient. Mild, moderate, and severe ischemia were defined as the involvement of 1–2, 3–5, and ≥6 myocardial segments, respectively (8).

### Cluster Analysis

A total of 44 clinical characteristics and CCTA/CMR imaging data were determined (Supplementary Material 7). The absence of collinearity between those 44 baseline variables was verified by principal component analysis (PCA). After the exclusion of collinear variables, 17 categorical variables were selected for the clustering model and the definition of phenogroups which were as follows: age, gender, obesity (body mass index ≥ 30 kg/m^2^), dyslipidemia, diabetes mellitus, hypertension, current or former smoker, family history of CAD, presence of typical angina, dyspnea on exertion, presence of atrial fibrillation (AF) on 12-lead ECG, history of peripheral arterial disease (PAD) defined by revascularization procedures involving the peripheral arterial circulation (15), presence of LV dilatation defined by LV end-diastolic volume indexed (LVEDVi > 100 ml/m^2^) (28), presence of LV systolic dysfunction defined by LV ejection fraction (LVEF) value <50% (28), ≥1 proximal segment with stenosis > 50%, rate of segments with non-calcified plaques ≥ 50%, the presence of LGE, and the presence of inducible ischemia. An unsupervised hierarchical clustering of principal components (HCPC function, from FactoMineR package, Vienna, Austria) algorithm was conducted using two steps: a multiple correspondence analysis (MCA) by which the principal components were obtained, followed by a hierarchical clustering analysis using Euclidean distance measures. Notably, the use of MCA was justified by the fact that all continuous variables included for clustering analysis were further classified into categorical variables. In addition, four dimensions were retained in the MCA output. The optimal number of clusters was determined based on the gain in within-inertia (inside group variance) and using the Nbclust package (Vienna, Austria). A detailed description of the used cluster analysis methods is provided in Supplementary Material 8.

### Statistical Analysis

Descriptive results were presented as percentages for categorical data and *M* ± *SD* or median [interquartile range (IQR)] for continuous variables, depending on the normality of their distribution. Comparisons between clusters were analyzed by ANOVA for numeric and chi-square test or Fisher exact test, as appropriate. The over- or under-representation of variables in each phenogroup were assessed by v-test, based on the hypergeometric distribution (HCPC function, from FactoMineR package, Vienna, Austria). Cox proportional hazards methods were used to assess the prognostic significance of each phenogroup and the prognostic value of inducible ischemia in each cluster. The assumption of the proportional HR was verified. The additional predictive value of phenogrouping for predicting MACE was calculated using Harrell's C-statistic increment, continuous net reclassification improvement (NRI), and the integrative discrimination index (IDI). A two-tailed *p*-value <0.05 was considered statistically significant. Statistical analysis and clustering were performed using R software, version 4.0.3 (R Project for Statistical Computing, Vienna, Austria).

## Results

### Patients Characteristics

The flowchart of the study is presented in Figure 1. Overall, 2,015 symptomatic patients with newly diagnosed obstructive CAD on CCTA and further referred for stress CMR completed the clinical follow-up and constituted our study cohort. The median (IQR) delay between CCTA and stress CMR was 9 (4-12) days. No patient had non-fatal MI, CV mortality, or death between CCTA and stress CMR. Among the 2,015 patients who were symptomatic at the time of CCTA and further referred to stress CMR, 2,001 (99.3%) had similar symptoms at the time of stress CMR. No severe adverse events occurred and detailed safety results are presented in Supplementary Material 9. Baseline patient characteristics and imaging data are shown in Table 1.

**Table 1:** Baseline and CCTA/CMR characteristics stratified by phenogroup.

	**All patients**	**Phenogroup 1**	**Phenogroup 2**	**Phenogroup 3**	***p*-value**
	**(*N* = 2,015)**	**(*N* = 854)**	**(*N* = 681)**	**(*N* = 480)**	
Age, years	70.0 ± 12.2	75.4 ± 10.3	66.8 ± 10.3	64.7 ± 11.1	**<0.001**
Males, *n* (%)	932 (46.3)	305 (35.7)	265 (38.9)	362 (75.4)	**<0.001**
Body mass index, kg/m^2^	28.3 ± 4.5	26.6 ± 3.6	31.3 ± 4.5	27.0 ± 3.8	**<0.001**
**Coronary risk factors**, ***n*** **(%)**					
Diabetes	637 (31.6)	116 (13.6)	426 (62.6)	95 (19.8)	**<0.001**
Hypertension	1,291 (64.1)	491 (57.5)	589 (86.5)	211 (44.0)	**<0.001**
Obesity^*^	516 (25.6)	73 (8.5)	384 (56.4)	59 (12.3)	**<0.001**
Dyslipidemia	988 (49.0)	328 (38.4)	480 (70.5)	180 (37.5)	**<0.001**
Smoking	422 (20.9)	91 (10.7)	85 (12.5)	246 (51.3)	**<0.001**
Family history of CAD	609 (30.2)	130 (15.2)	335 (49.2)	144 (30.0)	**<0.001**
**Medical history of CV disease**, ***n*** **(%)**					
History of PAD	134 (6.7)	72 (8.4)	35 (5.1)	27 (5.6)	0.022
Ischemic stroke	85 (4.2)	37 (4.3)	29 (4.3)	19 (4.0)	0.946
Pacemaker	8 (0.4)	3 (0.4)	3 (0.4)	2 (0.4)	1.000
**Symptoms the day of CCTA exam**, ***n*** **(%)**					
Symptomatic angina	1,351 (67.0)	625 (73.2)	470 (69.0)	256 (53.3)	**<0.001**
Dyspnea	664 (33.0)	229 (26.8)	211 (31.0)	224 (46.7)	**<0.001**
**Symptoms the day of stress CMR exam**, ***n*** **(%)**					
Symptomatic angina	1,349 (66.9)	623 (73.0)	470 (69.0)	256 (53.3)	**<0.001**
Dyspnea	652 (32.4)	223 (26.1)	208 (30.5)	221 (46.0)	**<0.001**
ESC risk Score, 10-year fatal CVD risk, (%)^†^	4.8 (1.1–6.0)	2.0 (0.3–3.6)	6.9 (1.9–9.2)	7.0 (2.0–10.1)	**<0.001**
**CCTA findings**					
No. of segments with any plaque or stenosis	3.3 ± 0.7	3.3 ± 1.0	3.5 ± 1.0	3.3 ± 0.8	**0.001**
No. of segments with stenosis >50%	1.2 ± 0.5	1.2 ± 0.5	1.2 ± 0.5	1.2 ± 0.6	1.000
No. of segments with stenosis >70%	0.1 ± 0.3	0.1 ± 0.3	0.1 ± 0.3	0.2 ± 0.4	**<0.001**
No. of proximal segments with stenosis >50%	0.2 ± 0.4	0.1 ± 0.3	0.2 ± 0.4	0.4 ± 0.4	**<0.001**
No. of proximal segments with stenosis >70%	0.1 ± 0.3	0.1 ± 0.2	0.1 ± 0.3	0.3 ± 0.3	**<0.001**
No. of segments with non-calcified plaques	1.1 ± 0.5	0.5 ± 0.5	1.1 ± 0.6	2.3 ± 1.1	**<0.001**
No. of segments with mixed plaques	1.1 ± 0.4	1.8 ± 0.4	0.6 ± 0.4	0.5 ± 0.5	0.891
No. of segments with calcified plaques	1.1 ± 0.4	1.0 ± 0.4	1.8 ± 0.4	0.5 ± 0.4	**<0.001**
No. of segments with calcified / mixed plaques	2.1 ± 0.4	2.8 ± 0.4	2.4 ± 0.4	1.0 ± 0.4	**<0.001**
**Number of vessels with obstructive CAD**					
1-vessel	1,894 (94.0)	831 (97.3)	643 (94.4)	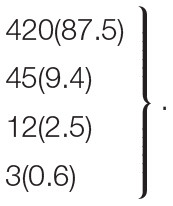	
2-vessel	79 (3.9)	11 (1.3)	23 (3.4)		**<0.001**
3-vessel	35 (1.2)	10 (1.2)	13 (1.9)		
LM	7 (0.3)	2 (0.2)	2 (0.3)		
**Stress CMR findings**					
**Cardiac rhythm during the CMR exam**, ***n*** **(%)**					
Sinus rhythm	1,938 (96.2)	833 (97.5)	644 (94.6)	461 (96.0)	0.182
Atrial fibrillation/supraventricular arrhythmia	77 (3.8)	21 (2.5)	37 (5.4)	19 (4.0)	0.061
LV ejection fraction, %	55.0 ± 10.1	58.1 ± 7.9	58.0 ± 8.3	45.1 ± 9.5	**<0.001**
LV end-diastolic volume index, ml/m^2^	80.7 ± 26.3	71.2 ± 18.0	70.7 ± 17.1	112.0 ± 25.4	**<0.001**
LV end-systolic volume index, ml/m^2^	37.5 ± 18.2	30.0 ± 10.5	30.1 ± 10.7	61.4 ± 16.8	**<0.001**
LV mass, g/m^2^	76.3 ± 9.8	72.3 ± 9.8	78.8 ± 9.6	79.7 ± 10.2	0.081
RV ejection fraction, %	61.6 ± 10.5	62.5 ± 10.5	61.4 ± 10.2	60.2 ± 10.2	0.58
Presence of LGE with is chemic pattern, *n* (%)	228 (11.3)	75 (8.8)	80 (11.7)	73 (15.2)	**0.002**
Presence of LGE with non-ischemic pattern, *n* (%)	41 (2.0)	10 (1.2)	12 (1.8)	19 (4.0)	**0.001**
Presence of viability if LGE with ischemic pattern, *n* (%)	135 (6.7)	48 (5.6)	44 (6.5)	43 (9.0)	**0.007**
Presence of inducible ischemia	302 (15.0)	106 (12.4)	78 (11.5)	118 (24.6)	**<0.001**
**CMR-related coronary revascularization**, ***n*** **(%)**	239 (11.9)	82 (9.6)	61 (9.0)	96 (20.0)	**<0.001**
by PCI	238 (11.8)	82 (9.6)	61 (9.0)	95 (19.8)	**<0.001**
by CABG	1 (0.05)	0 (0)	0 (0)	1 (0.2)	1.000

*Values are n (%), M ± SD or median (interquartile range)*.

* *Defined by BMI ≥ 30 kg/m^2^*.

*European score based on a modified SCORE project (https://www.escardio.org/Education/Practice-Tools/CVD-prevention-toolbox/SCORE-Risk-Charts) that did not take into account the total cholesterol level*.

*defined by the presence of LGE with <50% transmurality*.

*BMI, body mass index; CABG, coronary artery bypass graft; CAD, coronary artery disease; CCTA, coronary computed tomography angiography; CMR, cardiac magnetic resonance; CV, cardiovascular; CVD, cardiovascular disease; ECG, electrocardiogram; HF, heart failure; LGE, late gadolinium enhancement; LM, left main artery; LV, left ventricle; MI, myocardial infarction; PAD, peripheral atheroma disease; PCI, percutaneous coronary intervention; SD, standard deviation. The bold values indicates 2-tailed P-value reached statistical significance (P < 0.05)*.

Among the 2,015 patients (46.3% men, mean age 70 ± 12.2 years), 64.1% had hypertension, 49.0% had dyslipidemia, 31.6% had diabetes mellitus, 30.2% had a family history of CAD, 25.6% had obesity, and 20.9% were smokers. On CCTA, 94% of patients had 1-vessel obstructive CAD. Among the seven patients with LM stenosis > 50%, none had stenosis evaluated at > 70% in CCTA. By CMR, the study cohort had a mean LVEF of 55 ± 10%.

The presence of stress CMR inducible ischemia was detected in 302 (15%) patients with a mean extent of 2.6 ± 1.6 segments, and LGE was identified in 228 (11.3%) patients (Table 1).

Among 302 patients with ischemia, 287 (95%) underwent coronary angiography. Among those, 250 (82.7%) had obstructive CAD confirmed by invasive angiography and 239 (83.3%) underwent CMR-related coronary revascularization [238 (99.5%) PCI and 1 (0.5%) CABG]. Baseline patient characteristics and outcomes of patients with inducible ischemia are shown in Supplementary Material 10 according to the presence of obstructive CAD as defined by ICA.

### Clinical, CCTA, and CMR Characteristics of Each Phenogroup

**Figure 2:**
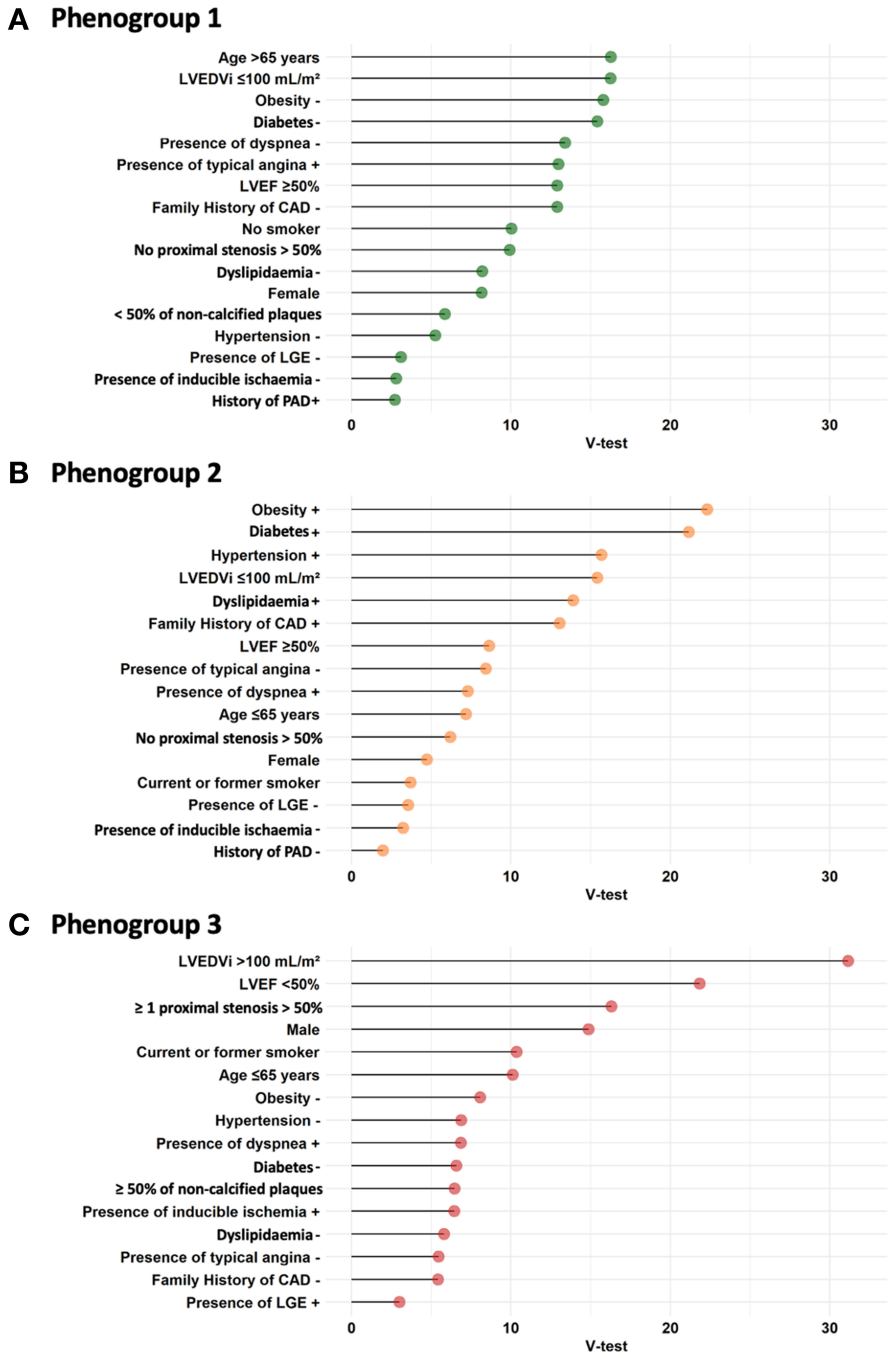
Characteristic plots of the three phenogroups [**(A)** Phenogroup 1, **(B)** Phenogroup 2, **(C)** Phenogroup 3]. The over- or under-representation of a variable within a phenogroup was analyzed by V-test within the hierarchical clustering, based on the hypergeometric distribution. The value of the v-test score indicates over-representation of this variable in the applicable phenogroup. Same abbreviations as Table 1.

**Figure 3:**
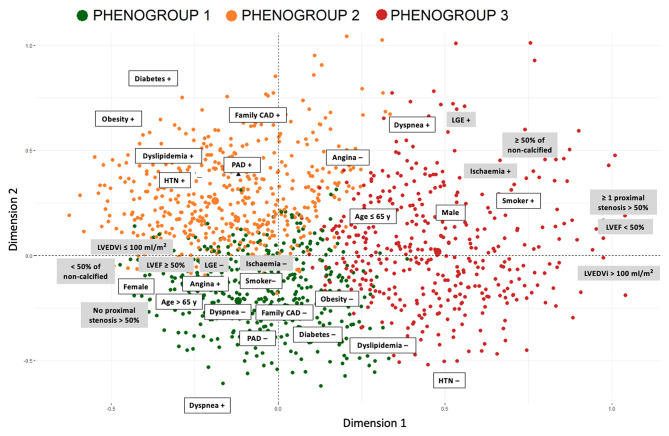
Cluster analysis. Biplot representation depicts the relationships between clinical characteristics (white box) and CCTA or CMR data (gray box) used for building phenogroups. Patients are displayed (dots) based on their individual characteristics. Results are projected onto the 2 first dimensions yielded by multiple correspondence analysis. Colors correspond to the very clear distinction of the 3 phenogroups from the cluster analysis (Supplementary Video 1). Same abbreviations as Table 1.

The results further showed that 20 clinical and CMR input variables had different contributions in defining the phenogroups (Figure 2). Hierarchical clustering established that three phenogroups yielded the highest gain in inertia (inside group variance) and was suggested by the HCPC function for k-means clustering (Supplementary Material 11). This led to the identification of three phenogroups (Figure 3) with significant differences in their clinical characteristics (Supplementary Material 12). Supplementary Video 1presented the three phenogroups according to the first three principal components in hierarchical clustering assessed on the three axes.

Phenogroup 1 (PG1; *n* = 854; 42.4%) included the oldest patients (75 ± 10 years), predominantly women (64%), with the lowest proportion of traditional risk factors (57% of hypertension, 38% of dyslipidemia, 14% of diabetes, 15% of family history of CAD, 11% of smoking, and 9% of obesity) compared with other patients. On CCTA, PG1 had the lowest proximal stenosis > 50% rate (6%) and the lowest proportion of non-calcified plaques (5% of patients with ≥ 50% of non-calcified plaques). On CMR, PG1 had the lowest LGE rate (9%) with a preserved mean LVEF value (58 ± 9%) and lower LV dilatation rate (5%).

Phenogroup 2 (PG2; *n* = 681; 33.8%) included younger patients (69 ± 10 years), predominantly women (61%), with the highest proportion of hypertension (86%), dyslipidemia (70%), diabetes (63%), obesity (56%), and family history of CAD (49%), but a lower rate of smokers (12%) compared with other patients. PG2 had the highest rate of calcified plaques (88%) on CCTA, with a preserved mean LVEF value (58 ± 8%) and the lowest LV dilatation rate (3%) on CMR.

Phenogroup 3 (PG3; *n* = 480; 23.8%) included the youngest patients (65 ± 11 years), predominantly men (75%), with the highest proportion of smokers (51%). PG3 had the highest proximal stenosis > 50% rate (40%) and the highest proportion of non-calcified plaques (18% of patients with ≥ 50% of non-calcified plaques). On CMR, PG3 had the highest LGE rate (15%) with the lowest mean LVEF value (45 ± 10%) and the highest LV dilatation rate (73%) (Supplementary Material 13).

It is worth noting that the association between the proportion of non-calcified plaques and the presence of LGE with ischemic pattern within each phenogroup is depicted in Supplementary Material 14.

### Association of Phenogroups With Outcomes

During a median follow-up of 6.8 (IQR 5.9–9.2) years, there was 277 (13.7%) MACE, including 149 (7.4%) CV mortality and 128 (6.4%) non-fatal MI. Furthermore, 184 all-cause mortality (9.1%) were recorded. Annualized event rates were 3.3% for MACE, 1.8% for CV mortality, and 1.9% for all-cause mortality.

**Figure 4:**
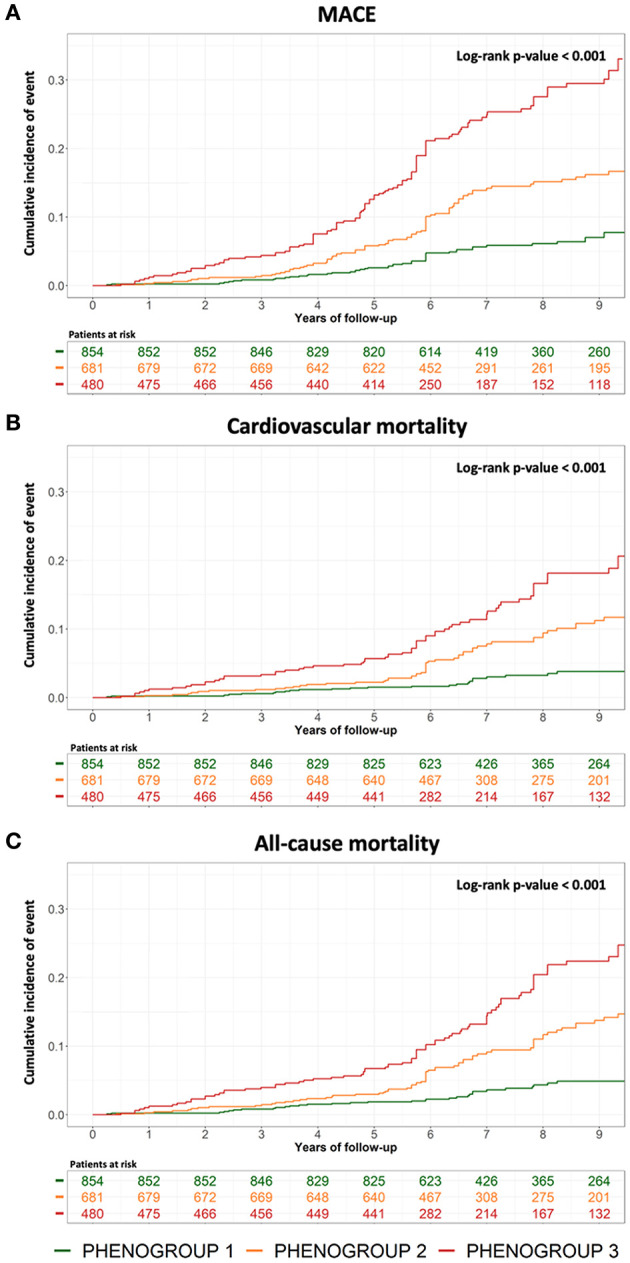
Kaplan-Meier curves for MACE **(A)**, cardiovascular mortality **(B)**, and all-cause mortality **(C)** of the three phenogroups.

Kaplan-Meier analysis showed significant differences for the occurrence of MACE across phenogroups, CV mortality, and all-cause mortality (all *p* < 0.001, Figure 4). PG1 has the lowest while PG3 has the highest risk of MACE (0.9 vs. 3.9 events per 100 patient-years; *p* < 0.001), CV mortality (0.4 vs. 2.1 events per 100 patient-years; *p* < 0.001), and all-cause mortality (0.5 vs. 2.6 events per 100 patient-years; *p* < 0.001) (Table 2). In multivariate Cox regression analysis, PG2 and PG3 were independently associated with a higher incidence of MACE compared with PG1 (HR: 1.98, 95% CI: 1.32–2.97 and HR: 2.15, 95% CI: 1.40–3.31, respectively, both *p* < 0.001) (Table 2; Supplementary Material 15). The addition of phenogroup characterization improved the prediction of MACE beyond clinical, CCTA and CMR predictors (C-statistic improvement: 0.04, *p* = 0.028; IDI = 0.033, *p* = 0.019; NRI = 0.21, *p* = 0.024) (Table 3). To note, the distribution of CCTA findings according to the occurrence of MACE in patients with ischemia is presented in Supplementary Material 16.

**Table 2:** Association of phenogroups with adverse outcomes on cox proportional hazard analysis.

	**Phenogroup 1**	**Phenogroup 2**	*p*-value^*^	**Phenogroup 3**	***p*-value^†^**
	**(*N* = 854)**	**(*N* = 681)**		**(*N* = 480)**	
**Outcomes**, ***n*** **(events/100 patient-years)**					
MACE	53 (0.9)	95 (2.1)	**<0.001**	129 (3.9)	**<0.001**
CV mortality	24 (0.4)	55 (1.9)	**<0.001**	70 (2.1)	**<0.001**
All-cause mortality	31 (0.5)	69 (1.5)	**<0.001**	84 (2.6)	**<0.001**
**Unadjusted HR (95% CI)**					
MACE	1.0	2.37 (1.70–3.32)	**<0.001**	5.08 (3.68–6.99)	**<0.001**
CV mortality	1.0	3.01 (1.86–4.86)	**<0.001**	5.80 (3.65–9.23)	**<0.001**
All-cause mortality	1.0	2.92 (1.91–4.47)	**<0.001**	5.38 (3.56–8.13)	**<0.001**
**Adjusted HR (95% CI)^‡^**					
MACE	1.0	1.98 (1.32–2.97)	**<0.001**	2.15 (1.40–3.31)	**<0.001**
CV mortality	1.0	2.27 (1.30–3.96)	**<0.001**	2.31 (1.24–4.29)	**<0.001**
All-cause mortality	1.0	2.10 (1.28–3.44)	**0.003**	2.20 (1.26–3.83)	**0.005**

* *The comparisons between PG1 and PG2 that were statistically significant with p <0.05 are shown in bold type*.

† *The comparisons between PG1 and PG3 that were statistically significant with p <0.05 are shown in bold type*.

‡ *Covariates in the model by stepwise variable selection with entry and exit criteria set at the p ≤ 0.1 level: age, male, hypertension, diabetes, dyslipidemia, family history of CAD, body mass index, smoker status, LVEF per 10%, presence of LGE, presence of ischemia, presence of ≥1 proximal stenosis > 50% and proportion of non-calcified plaques > 50%*.

*CI, confidence interval; CV, cardiovascular; HR, hazard ratio; MACE, major adverse cardiac events; PG, phenogroup*.

**Table 3:** Discrimination and reclassification of the traditional model with and without phenogroups for prediction of MACE.

	Traditional model^*^	**Traditional + phenogrouping model^†^**	***p*-value**
**Discrimination**			
C-index (95% CI)	0.80 (0.73–0.86)	0.84 (0.77–0.88)	*p* = 0.028
IDI (95% CI)	Reference	0.033 (0.012–0.057)	*p* = 0.019
**Reclassification**			
NRI (95% CI)	Reference	0.210 (0.136–0.340)	*p* = 0.024

* *Covariates in the traditional model by stepwise variable selection with entry and exit criteria set at the p ≤ 0.1 level: age, male, hypertension, diabetes, dyslipidemia, family history of CAD, body mass index, smoker status, LVEF per 10%, presence of LGE, presence of ischemia, presence of ≥1 proximal stenosis > 50% and proportion of non-calcified plaques > 50%*.

† *Covariates in the traditional + phenogrouping model: traditional model with the variable “phenogrouping” defined according to the PG of each patient (PG1, PG2, or PG3)*.

*Abbreviations same as in Table 2. LR, likelihood ratio; IDI, integrative discrimination index; NRI, net reclassification improvement*.

### Prognostic Value of Inducible Ischemia and Proximal Non-calcified Plaques in Each Phenogroup

**Figure 5:**
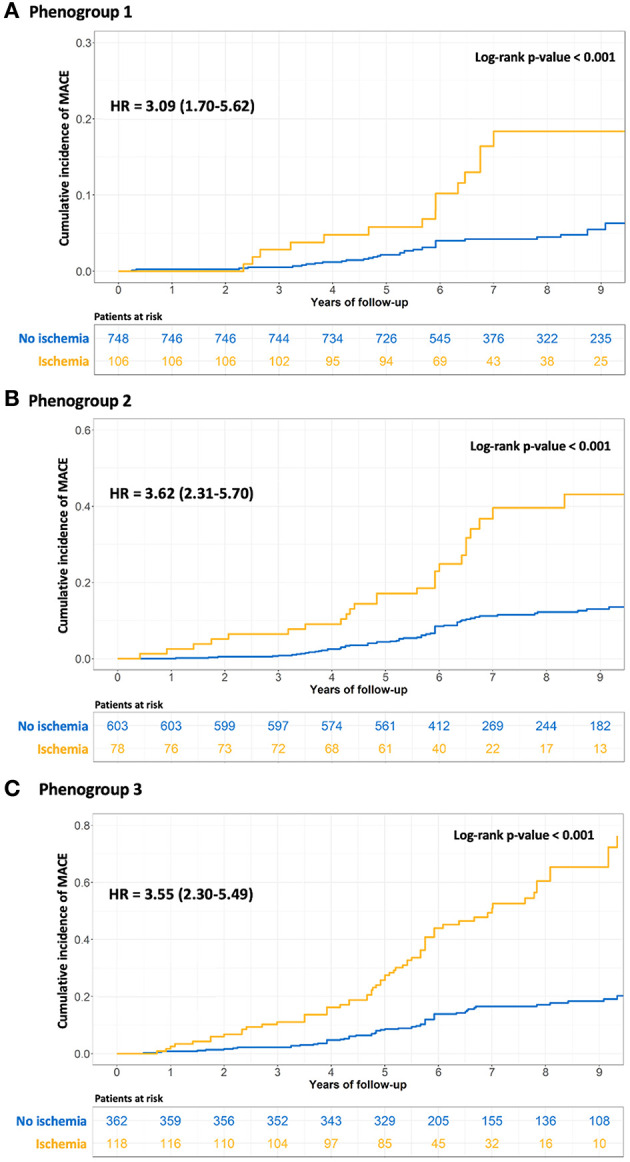
Kaplan-Meier curves for MACE in each phenogroup stratified by inducible ischemia on stress CMR. The univariable analysis for MACE was performed using the log-rank test to compare patients with and without inducible ischemia in phenogroup 1 **(A)**, phenogroup 2 **(B)**, and phenogroup 3 **(C)**.

In each PG, the presence of inducible ischemia by CMR was associated with the occurrence of MACE (PG1, HR = 3.09, 95% CI, 1.7–5.62; PG2, HR = 3.62, 95% CI, 2.31–5.7; PG3, HR = 3.55, 95% CI, 2.3–5.49; all *p* < 0.001; Figure 5). Using CCTA data, the number of proximal segments with non-calcified plaques was associated with the occurrence of MACE within each PG (PG1, HR = 1.57, 95% CI, 1.31–1.89; PG2, HR = 1.73, 95% CI, 1.22–2.45; PG3, HR = 2.06, 95% CI, 1.63–2.53; all *p* < 0.001; Supplementary Material 17).

## Discussion

In this cohort of consecutive patients with newly diagnosed CAD by CCTA and further referred for functional assessment by stress CMR, an unsupervised approach of hierarchical clustering integrating clinical, CCTA, and CMR data identified three mutually exclusive phenogroups of patients. These phenogroups were associated with distinct clinical, CAD burden, and prognostic profiles. Furthermore, phenogrouping had an incremental prognostic value for predicting MACE above clinical, CCTA, and CMR predictors. The three phenogroups integrated a broad range of clinical and CV imaging variables: (PG1) older patients with very few traditional risk factors, non-calcified plaques on CCTA, and preserved LVEF; (PG2) younger women with a metabolic syndrome profile including traditional risk factors (29), except smoking, calcified plaques on CCTA, and a preserved LVEF; (PG3) younger men smokers with proximal non-calcified plaques on CCTA and myocardial scar causing systolic dysfunction associated with LV dilatation. While the presence of inducible ischemia by CMR and the number of proximal segments with non-calcified plaques by CCTA were involved in the clustering method, both remained associated with the occurrence of MACE within each phenogroup.

These findings suggested that each phenogroup might represent a more homogeneous subset of CAD patients with similar atheromatous pathophysiology and risk profile. The characteristics of these three phenogroups might raise some hypotheses explaining the pathogenesis of the atheromatous plaque in those consecutive patients. The data suggested that age might not be sufficient to accurately stratify the risk, since elderly patients with CAD (PG1) showed better outcomes than younger patients with a metabolic syndrome profile (PG2). Moreover, despite patients of PG3 portended a higher risk, patients in this group were younger, suggesting a potential benefit of CCTA for early detection of CAD. Consistently, the findings that younger men smokers with non-calcified proximal plaques on CCTA (PG3) were at higher risk which suggested the use of more aggressive interventions for improved prevention of MACE in those patients. Two different profiles of coronary plaque composition by CCTA were highlighted: elderly women at high CV risk with metabolic syndrome and calcified plaques (PG2); young men smokers with non-calcified plaques (PG3). Prior reports using clustering analysis in patients with hypertension showed that young men smokers constituted the phenogroup with the most severe carotid artery disease (30). Consistently, PG3 presented the most severe CAD with the worse outcome. The identification of subsets of patients with distinct clinical, CAD burden profiles, and outcomes could help guide future clinical trials, especially for very high-risk patients. Recent studies had shown promising new therapies targeting inflammation and coagulation to improve outcomes (31, 32).

The prevalence of inducible ischemia (15%) and LGE (11%) were consistent with previous large studies in patients referred to stress CMR for suspected or known CAD (8, 9). This prevalence might appear relatively low in patients with obstructive CAD on CCTA, but it could be explained by the frequent overestimation of the severity of coronary stenosis by CCTA (33). The rate of MACE reported over the follow-up period (13.7%) was in line with contemporary stress CMR cohorts (8) and the ISCHEMIA trial (34), and was higher than that described in patients with normal CMR (1%/year) (8).

The identification of phenogroups offered incremental prognostic value above a final model including traditional CV risk factors, CCTA, and CMR data, showing the potential impact of unsupervised approaches to better stratify these patients. How these findings could lead to therapeutic implications deserve further investigation.

### Study Limitations

Although patients were included prospectively, the study design was retrospective with 7.8% of patients lost to follow-up. Baseline data for medications were not collected. In the absence of contraindications, all patients with obstructive CAD on CCTA received optimal medical treatment, including statins, as recommended by current guidelines (2). Although a recent study has emphasized the effects of statins on plaque composition (35), the detailed medical regimen was not prospectively collected and this question was beyond the scope of the study. Symptoms were assessed by the sole presence of symptomatic angina or dyspnea on exertion without standardized classification. Patients with a high-grade > 90% stenosis on CCTA were referred for invasive coronary angiography and excluded from analysis, limiting the extrapolation of the findings in this group of patients. The exclusion of patients with moderate to severe renal failure limited the extrapolation of results to a general patient population. The analysis of CMR scans was visual, which represented the most widely accepted clinical method with optimal diagnostic accuracy. The coronary artery calcium score was not systematically performed before CCTA in symptomatic patients. Although CCTA protocol followed current guidelines, specific and quantitative plaque analysis might be affected. In addition, the quantitative assessment of the low-attenuation plaques by CCTA was not systematically performed in this study. Dipyridamole was used as a stress agent mainly because of medico-economic reasons and a very close efficacy/safety profile compared to adenosine. The current analysis did not address the question of whether unsupervised learning based on CCTA alone could predict outcomes or functionally significant CAD as assessed by stress CMR. The current study was not designed to assess which stress CMR or CCTA parameter was most powerful in predicting MACE. Although recent studies have highlighted the role of biomarkers such as Troponin T in risk stratification (36), these data were not available in this study. Although the current approach might represent a major shift from traditional studies, it was a novel attempt toward a more personalized approach to patients with high CV risk. Whether automated unsupervised phenogrouping of CAD patients could improve clinical decision-making should be further explored in prospective studies.

## Conclusion

Using automated unsupervised cluster analysis, three different phenogroups of patients with newly diagnosed CAD by CCTA were identified and associated with significant differences in clinical presentation, CCTA, CMR data, and outcomes. Although inducible ischemia and proximal non-calcified plaques were involved in the clustering method, wherein both remained associated with the occurrence of MACE within each phenogroup. Further prospective studies should evaluate how these data using automated unsupervised phenogrouping could impact clinical decision-making and guide therapy.

## Data Availability Statement

The raw data supporting the conclusions of this article will be made available by the authors, without undue reservation.

## Ethics Statement

The studies involving human participants were reviewed and approved by Local Ethics Committee. The patients/participants provided their written informed consent to participate in this study.

## Author Contributions

TP, AA, and JG conceived the study design. TP, FS, TH, SC, TU, PG, and JG obtained CMR images and analyzed CMR scans. TP, AA, and JG analyzed data and drafted the manuscript with critical revision. JG and ST have technically defined the CMR protocol. All authors participated in the discussion of the concept of the study, read, and approved the final manuscript.

## Conflict of Interest

ST is an Engineer at Siemens Healthineers, France. She is in charge of fine tuning of CMR sequences in the Hôpital Privé Jacques Cartier, Massy, France. The remaining authors declare that the research was conducted in the absence of any commercial or financial relationships that could be construed as a potential conflict of interest.

## Publisher's Note

All claims expressed in this article are solely those of the authors and do not necessarily represent those of their affiliated organizations, or those of the publisher, the editors and the reviewers. Any product that may be evaluated in this article, or claim that may be made by its manufacturer, is not guaranteed or endorsed by the publisher.

## Abbreviations

AF: atrial fibrillation
CABG: coronary artery bypass grafting
CAD: coronary artery disease
CMR: cardiovascular magnetic resonance
HF: heart failure
LGE: late gadolinium enhancement
LV: left ventricular
LVEDVi: left ventricular end-diastolic volume indexed
LVEF: left ventricular ejection fraction
MACE: major adverse clinical events
MI: myocardial infarction
PAD: peripheral arterial disease
PCI: percutaneous coronary intervention
RV: right ventricle.
