# Supplementary material for: Phenotypic Clustering of Patients With Newly Diagnosed Coronary Artery Disease Using Cardiovascular Magnetic Resonance and Coronary Computed Tomography Angiography
Source: Front Cardiovasc Med. 2021 Nov 18;8:760120. doi: 10.3389/fcvm.2021.760120 (PMC8636934; doi:10.3389/fcvm.2021.760120)
Supplement: Supplementary file 1 [file Data_Sheet_1.docx]

**SUPPLEMENTAL MATERIAL**

**Table of Contents:**

- Supplemental Material 1: List of exclusion criteria.
- Supplemental Material 2: Definition of clinical events.
- Supplemental Material 3: CCTA protocols.
- Supplemental Material 4: Radiation exposure.
- Supplemental Material 5: CMR protocol and analysis.
- Supplemental Material 6: CMR sequence parameters.
- Supplemental Material 7: Clinical, CCTA and stress CMR variables included in the clustering model.
- Supplemental Material 8: Cluster analysis methods.
- Supplemental Material 9: Stress CMR safety results.
- Supplemental Material 10: Baseline Clinical characteristics and Outcomes of patients inducible ischemia according to the presence of obstructive CAD as defined by ICA.
- Supplemental Material 11: Determination of the optimal number of phenogroups.
- Supplemental Material 12: Phenogroups characteristics.
- Supplemental Material 13: Box plots: Distribution of Cardiovascular Magnetic Resonance Parameters.
- Supplemental Material 14: Association between the proportion of non-calcified plaques and the presence of LGE with ischemic pattern within each phenogroup.
- Supplemental Material 15: Sensitivity analysis: Association of phenogroups with adverse outcomes on Cox proportional hazard analysis.
- Supplemental Material 16: Distribution of CCTA findings according to the occurrence of MACE in patients with ischemia.
- Supplemental Material 17: Association of the number of proximal segments with noncalcified plaques and the occurrence of MACE.

**SUPPLEMENTAL MATERIAL 1**

**List of exclusion criteria**

- Previously known CAD before index CCTA was defined as: prior myocardial infarction (MI), known significant coronary stenosis, previous coronary revascularization, or presence of any stents or grafts/graft stenosis as recorded by CT findings.
- CCTA exclusion criteria were pregnancy, glomerular filtration rate <60 ml/min/1.73 m^2^, and known allergy to contrast agents.
- Of patients with obstructive CAD on CCTA and referred for stress CMR, exclusion criteria were: (i) contraindication to CMR (cerebral clips, metallic eye implant); (ii) contraindication to dipyridamole (severe asthma or chronic obstructive pulmonary disease, second- or third-degree atrioventricular block); (iii) known cardiomyopathy (e.g. hypertrophic, dilated, or infiltrative) and acute or chronic myocarditis; (iv) known allergy to gadolinium-based contrast medium; and (v) glomerular filtration rate <30 mL/min/1.73 m^2^.

**SUPPLEMENTAL MATERIAL 2**

**Definition of all clinical events**

Nonfatal MI was defined by typical angina of ≥ 20 min duration, ECG changes, and a rise in troponin or creatine kinase level above the 99 percentile of the upper reference limit(1). Cardiovascular mortality was defined as sudden cardiac death with documented fatal arrhythmias or any death immediately preceded by acute MI, acute or exacerbation of heart failure, or stroke. All-cause mortality was defined using the electronic French National Registry of Death (*Institut National de la Statistique et des Etudes Economiques*, INSEE registry). All clinical events were defined according to standardized definitions(2). For patients who underwent PCI within 90 days after the index examination, peri-procedural events (MI or cardiovascular mortality)(3) were not included in the analysis.

References

1. Thygesen K, Alpert JS, Jaffe AS, Chaitman BR, Bax JJ, Morrow DA, et al. Fourth Universal Definition of Myocardial Infarction (2018). Circulation. 2018 13;138(20):e618–51.

2. Hicks KA, Tcheng JE, Bozkurt B, Chaitman BR, Cutlip DE, Farb A, et al. 2014 ACC/AHA Key Data Elements and Definitions for Cardiovascular Endpoint Events in Clinical Trials. J Am Coll Cardiol. 2015;66(4):403–69.

3. Stone GW, Ben-Yehuda O, Sabik JF, Kappetein AP, Serruys PW. Considerations for an optimal definition of procedural myocardial infarction. Eur Heart J. 2020;41(17):1704–5.

**SUPPLEMENTAL MATERIAL 3**

**Coronary computed tomography angiography (CCTA) Protocols**

***Patient preparation before CCTA***

Prior to CCTA, in patients with a resting heart rate (HR) >65 beats/min, b-blocker therapy (atenolol) was administered intravenously, with a titrated dose of up to 50 mg to achieve a target HR of ≤65 beats/min. All patients received sublingual nitrates to ensure coronary vasodilation before the scan. A bolus of 60 to 110 ml nonionic contrast agent (Optiray 350, Tyco Healthcare, Montreal, Quebec, Canada; or Iomeprol 350 (350 mg/ml, Iomeron, Bracco, Italy) or Iobitridol (Xenetix 350, Guerbet, Aulnay Sous Bois, France) was injected at a rate of 4 ml/s when body weight was <60 kg, or 5 ml/s when body weight was >60 kg, followed by a 20 ml saline flush. All scans were performed using a bolus tracking technique with visual assessment to determine the precise timing of image acquisition.

***Imaging Protocols***

***A)*** Between 2008-2011, CCTA acquisitions were performed using a 64-slice multidetector computed tomography (MDCT) scanner (Brilliance 64, Philips Healthcare), which contains single source, 64 detector rows. Retrospective electrocardiogram (ECG)-gated 64-slice CCTA was performed with the following typical scan parameters: 64 x 0.625 mm collimation, 400 ms rotation time, pitch 0.2, 120 kVp tube voltage, and effective tube current-time product (normalized to pitch) 800-1000 mAs.

***B)*** Between 2012-2018, CCTA acquisitions were performed using a 256-slice MDCT scanner (Brilliance iCT, Philips Healthcare), which contains single source, 128 detector rows with focal spot-shift technology. Retrospective ECG-gated 256-slice CCTA was performed with the following typical scan parameters: 128 x 2 x 0.625 mm collimation, 270 ms rotation time, pitch of 0.16, 120 kVp tube voltage, and effective tube current-time product (normalized to pitch) 800-1000 mAs.

Between 2008 and 2018, the standard temporal resolution (210 ms for the 64-slice MDCT and 135 ms for the 256-slice MDCT) was improved through the use of advanced cardiac multi-cycle reconstruction algorithms that combined data from consecutive cardiac cycles.^1^ Additionally, the overlapped pitch, along with the use of cardiac gating algorithms (Beat-to-Beat Variable Delay Algorithm, Philips Healthcare) enabled the detection and reconstruction of the same physiological cardiac phase of interest (for example, quiescent phase corresponding to ventricular diastasis).^2^

***C)*** Between 2019 and 2020, CCTA acquisitions were performed using a 320-slice MDCT scanner (Aquilion One Genesis; Canon Medical Systems) with prospectively ECG-triggered single heartbeat acquisition protocol. Images were acquired with the typical following parameters: 320-detector rows x 0.5-mm detector element width, 16-cm coverage, 100 kV tube voltage, and a 350 ms gantry rotation time at a single focal spot, with the best temporal resolution of 175 ms using half-scan reconstruction. This configuration allowed three-dimensional (3D) volumetric whole-heart imaging during the diastole of one R-R interval.

References:

1. Manzke R, Grass M, Nielsen T, Shechter G, Hawkes D. Adaptive temporal resolution optimization in helical cardiac cone beam CT reconstruction. Med Phys. 2003;30(12):3072–80.

2. Vembar M, Garcia MJ, Heuscher DJ, Haberl R, Matthews D, Böhme GE, et al. A dynamic approach to identifying desired physiological phases for cardiac imaging using multislice spiral CT. Med Phys. 2003;30(7):1683–93.

**SUPPLEMENTAL MATERIAL 4**

**Radiation exposure**

The dose-length product (DLP), defined as the total radiation energy absorbed by the patient’s body, was individually reported from each CCTA in mSv/mGy.cm. The effective radiation dose was calculated as the product of the dose-length product times a conversion coefficient for the chest (K = 0.014 mSv/mGy·cm).

The mean ± standard deviation DLP was 739 ± 197 mGy/cm, which is equivalent to 10.3 ± 2.8 mSv for Brilliance 64 CT (Philips Healthcare, between 2008-2011); 228.6 ± 78.1 mGy/cm, which is equivalent to 3.2 ± 1.1 mSv for Brilliance iCT (Philips Healthcare, between 2012-2018); and 57.1 ± 10.7 mGy/cm, which is equivalent to 0.80 ± 0.15 mSv for Aquilion One Genesis (Canon Medical Systems, between 2019 and 2020).

**SUPPLEMENTAL MATERIAL 5**

**Cardiovascular magnetic resonance (CMR) Protocol**

CMR was performed in a dedicated cardiovascular MR laboratory on a 1.5T scanner (MAGNETOM Espree from December 2008 to August 2015 and MAGNETOM Aera from August 2015 to January 2020, Siemens Healthcare, Erlangen, Germany) with an 18-channel anterior phased-array coil. Long-axis (2-, 3-, and 4-chamber) and short-axis cine images encompassing the left ventricle from base to apex were obtained with a segmented retrospectively gated balanced steady-state free-precession (b-SSFP) sequence. Vasodilation was induced with dipyridamole injected at 0.84 mg/kg over 3 min. Then, a bolus of gadolinium-based contrast agent (Dotarem^®^, Guerbet, France, 0.1 mmol/kg) was injected at a rate of 5.0 ml/s with an injector (Mallinckrodt Optistar^®^ Elite). Stress perfusion imaging was performed using an ECG-triggered saturation-prepared b-SSFP sequence. A series of six slices (four short-axis views, a 2-chamber, and a 4-chamber view) were acquired every other heartbeat. Then, theophylline was injected intravenously to null the effect of dipyridamole. Ten minutes after contrast injection, breath-hold contrast-enhanced 3D T1-weighted inversion-recovery gradient-echo sequences were acquired to detect late gadolinium enhancement (LGE). The inversion time was individually adjusted to null normal myocardium. Patients were asked to refrain from caffeine at least 12 h before CMR. Safety was studied with clinical monitoring 1 h after CMR. A 12-lead ECG was performed both before and after CMR examination.

The *syngo*.via software (Siemens Healthcare, Erlangen, Germany) was used for image display and processing, and Hemolia (Clinigrid Inc., Paris, France) was used for data storage and reporting.

**SUPPLEMENTAL MATERIAL 6**

**Table. CMR sequence parameters.**

|  | **Cine**  **long-axis** | **Perfusion** | **Cine**  **short-axis** | **TI Scout** | **LGE** |
| --- | --- | --- | --- | --- | --- |
| **Pulse sequence** | b-SSFP | Saturation-recovery b-SSFP | b-SSFP | b-SSFP | 3D inversion-recovery fast gradient echo (FGRE) |
| **Field of view (FoV)** | 380 x 340 mm² | 370 x 314 mm² | 380 x 304 mm² | 380 x 307 mm² | 340 x 340 mm² |
| **Slice thickness** | 6 mm | 8 mm | 8 mm | 8 mm | 6 mm |
| **Matrix size** | 256 pixels | 224 pixels | 240 pixels | 208 pixels | 272 pixels |
| **Phase resolution** | 95 % | 80 % | 95 % | 100 % | 89 % |
| **Slice resolution** | NA | NA | NA | NA | 67 % |
| **Phase oversampling** | 0 % | 0 % | 0 % | 0 % | 20 % |
| **Slice oversampling** | NA | NA | NA | NA | 20 % |
| **Voxel size (reconstructed)** | 1.5 x 1.5 x 6 mm^3^ | 1.7 x 1.7 x 8 mm^3^ | 1.6 x 1.6 x 8 mm^3^ | 1.8 x 1.8 x 8 mm^3^ | 1.3 x 1.3 x 6 mm |
| **Voxel size (acquired)** | 1.5 x 1.6 x 6 mm^3^ | 1.7 x 2.0 x 8 mm^3^ | 1.6 x 1.7 x 8 mm^3^ | 1.8 x 1.8 x 8 mm^3^ | 1.3 x 1.4 x 8 mm |
| **TE/TR** | 1.18/2.9 ms | 1.04/2.8 ms | 1.15/2.8 ms | 1.31/3 ms | 1.35/3.4 ms |
| **Flip angle (FA)** | 55° | 70° | 55° | 30° | 9° |
| **Acceleration** | 6.1  (CS) | 2  (GRAPPA) | 6.5  (CS) | 4.2  (CS) | 2  (GRAPPA) |

Abbreviations: bSSFP: balanced steady-state free-precession; CS: compressed-sensing; FGRE: fast gradient echo; FoV: reconstructed field of view; LGE: late gadolinium enhancement; TE: echo time; TI: inversion time; TR: repetition time, NA: not applicable.

**SUPPLEMENTARY FILE 7**

**Table. Clinical, CCTA and stress CMR variables included in the clustering model.**

| **Phenotypic domain** | **Clinical, CCTA and stress CMR variables** |
| --- | --- |
| **Demographics** | Age^*^, gender^*^. |
| **Cardiovascular risk factors** | Hypertension^*^, body mass index^*^, diabetes mellitus^*^, dyslipidemia^*^, current or previous smoking^*^ and family history of coronary artery disease^*^. |
| **Medical history** | Ischemic stroke, pacemaker and peripheral atheroma disease^*^. |
| **Physical characteristics** | Typical symptomatic angina^*^, dyspnea on exertion^*^ and body surface area. |
| **Electrocardiogram** | Heart rate, cardiac rhythm (sinus or atrial fibrillation). |
| **CCTA parameters** | No. of segments with any plaque or stenosis, with stenosis >50%, and with stenosis >70%.  No. of proximal segments with stenosis >50%^*^ and with stenosis >70%.  No. of segments with non-calcified plaques^*^, with mixed plaques, with calcified plaques and with calcified or mixed plaques.  Number of vessels with obstructive CAD. |
| **Stress CMR parameters** | LV mass index, LV end-diastolic volume index^*^, LV end-systolic volume index, LV ejection volume index, LV ejection fraction^*^, presence of LGE^*^, presence of viability, presence of inducible ischemia, RV end-diastolic volume index, RV end-systolic volume index and RV ejection fraction. |
|  |  |

* Clinical and CMR variables used in the clustering model.

*Abbreviations: CMR: cardiovascular magnetic resonance; LV: left ventricular; RV: right ventricular.*

**SUPPLEMENTARY FILE 8**

**Cluster analysis methods.**

***Data processing***

Phenogroups were defined based on 17 categorical variables, among baseline, clinical CCTA and stress CMR findings: age, gender, obesity (body mass index ≥30 kg/m²), dyslipidaemia, diabetes mellitus, hypertension, current or former smoker, family history of CAD, presence of typical angina, dyspnea on exertion, presence of atrial fibrillation (AF) on 12-lead ECG, history of peripheral arterial disease (PAD) defined by revascularization interventions involving the peripheral arterial circulation,^11^ presence of LV dilatation defined by LV end-diastolic volume indexed (LVEDVi >100 mL/m²),^28^ presence of LV systolic dysfunction defined by LV ejection fraction (LVEF) value <50%,^28^ ≥ 1 proximal segment with stenosis >50%, rate of segments with non-calcified plaques ≥ 50%, the presence of LGE, and the presence of inducible ischemia.

***Unsupervised machine learning methods to define phenogroups***

We used the optCluster package to determine the optimal clustering method. The optCluster results indicated the diana method as the most robust on validation parameters. All other clustering methods were overall quite similar in performance. Phenotypic clusters were defined using an unsupervised hierarchical clustering of principal components (HCPC) approach. Indeed, we used the FactoMineR package using two steps: a Multiple Correspondence Analysis (MCA), by which principles components were obtained, following by a hierarchical clustering analysis (using Euclidean distance measures).

The optimal number of phenogroups was determined based on the gain in within-inertia (inside group variance), the shape of the dendrogram tree and using the Nbclust package. After cutting the tree to the desired number of clusters, a k-means consolidation was performed to increase the robustness of the created clusters, which is included in the HCPC method by default.

**SUPPLEMENTAL MATERIAL 9**

**Stress CMR safety results**

There were 16 cases of unstable angina and 2 acute pulmonary edemas, but no transient ischemic attack, disabling stroke, ST elevation MI or sustained ventricular tachycardia were recorded in relation to stress CMR. Among the 2,210 patients who completed the stress CMR protocol, the main adverse events during or immediately after the study were: 289 headaches (13.1%), 209 nausea or vomiting (9.4%), 131 chest discomfort due to dipyridamole (5.9%), 78 dizziness (3.5%) and 38 anginas with ECG evidence of myocardial ischemia (1.7%). For all patients, symptoms resolved quickly with intravenous theophylline and additional sublingual nitrates and/or intravenous betablockers in 28 patients (1.3%).

**SUPPLEMENTARY FILE 10**

**Table. Baseline Clinical characteristics and Outcomes of patients inducible ischemia according to the presence of obstructive CAD as defined by ICA.**

|  | **Ischemia with obstructive CAD as defined by ICA**  **(N=250)** | **Ischemia without obstructive CAD as defined by ICA (N=37)** | **p value** |
| --- | --- | --- | --- |
|  |  |  |  |
| Age, years | 72.0 ± 11.5 | 72.3 ± 11.0 | 0.57 |
| Males, n (%) | 182 (72.8) | 25 (68.0) | 0.08 |
| Body mass index, kg/m² | 27.4 ± 4.3 | 28.0 ± 6.2 | 0.39 |
|  |  |  |  |
| **Coronary risk factors**, n (%) |  |  |  |
| Diabetes mellitus | 82 (32.8) | 12 (32.4) | 0.76 |
| Hypertension | 164 (67.6) | 27 (73.0) | 0.031 |
| Obesity^*^ | 38 (15.2) | 23 (62.1) | <0.001 |
| Dyslipidemia | 135 (54.0) | 18 (48.6) | 0.06 |
| Smoking | 54 (21.6) | 10 (27.0) | 0.019 |
|  |  |  |  |
| **Medical history of CV disease**, n (%) |  |  |  |
| Peripheral atheroma | 20 (8.0) | 6 (16.2) | 0.081 |
| Ischemic stroke | 7 (2.8) | 10 (27.0) | <0.001 |
| Pacemaker | 1 (0.4) | 0 (0) | 1.000 |
| Renal failure^†^ | 2 (0.8) | 1 (26.4) | 0.091 |
|  |  |  |  |
| **Outcomes** |  |  |  |
| MACE (n=105) | 94 (37.6) | 11 (29.7) | 0.001 |
| Cardiovascular mortality (n=57) | 51 (20.4) | 6 (16.2) | 0.021 |
| All-cause mortality (n=74) | 66 (26.4) | 8 (21.6) | 0.083 |
|  |  |  |  |

*Values are n (%), mean ± SD, or median (interquartile range).*

* defined by BMI ≥30 kg/m2

^†^ defined by glomerular filtration rate between 60 and 90 ml/min/1.73 m^2^.

*Abbreviations: BMI: body mass index; CABG: coronary artery bypass graft; CAD: coronary artery disease; CCTA: coronary computed tomography angiography; CMR: cardiac magnetic resonance; CV: cardiovascular; CVD: cardiovascular disease; ECG: electrocardiogram; ICA: invasive coronary angiography;* *MACE: major adverse cardiac events; SD: standard deviation.*

**SUPPLEMENTARY FILE 11**

**Figure. Determination of the optimal number of phenogroups.**

Hierarchical clustering for the identification of the optimal number of phenogroups. Three phenogroups yielded the highest gain in inertia (inside group variance) and was suggested by the HCPC function for k-means clustering (Panel A).

The dendrogram representation of the three phenogroups is presented in Panel B.

**
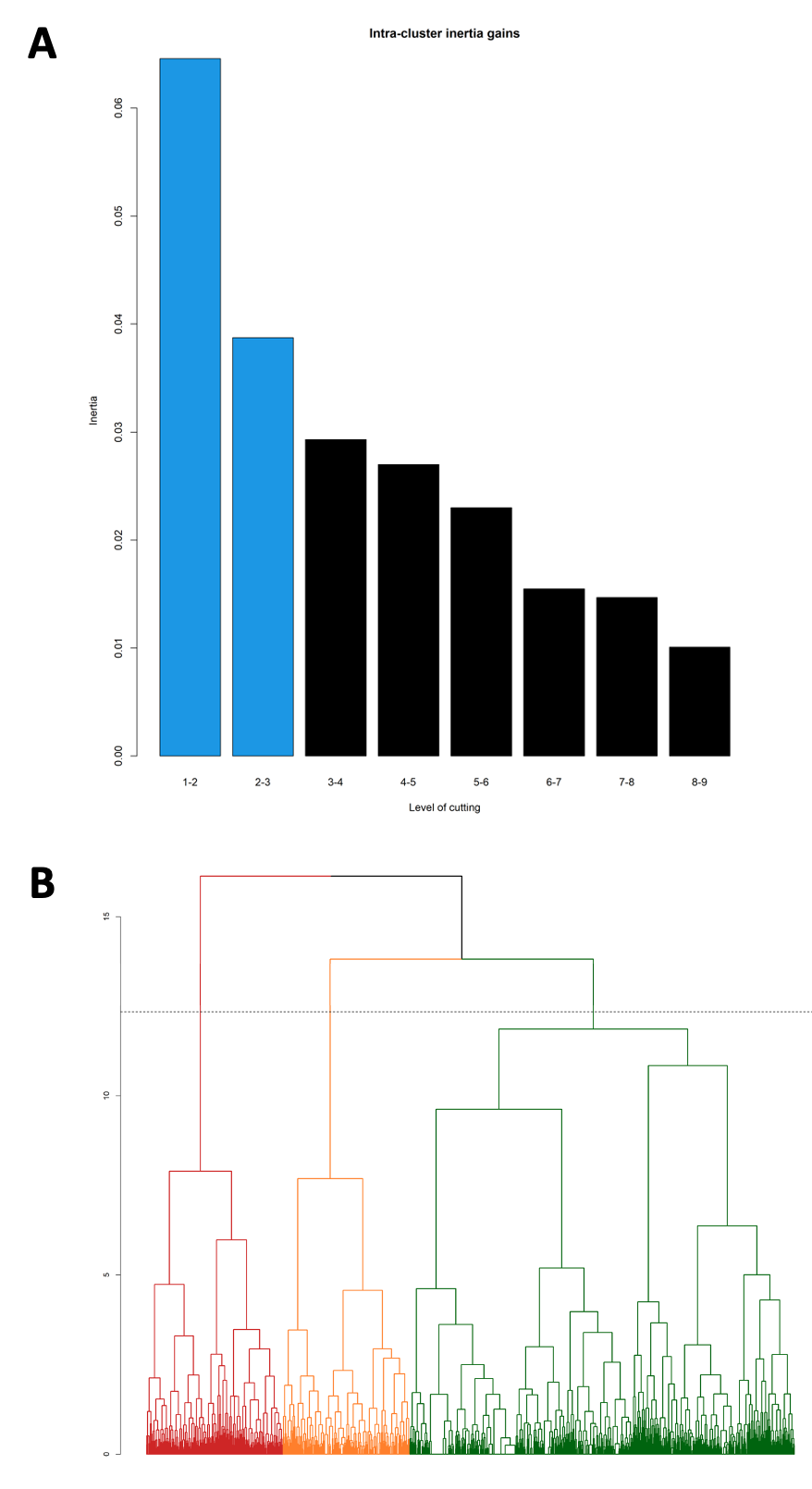
**

**SUPPLEMENTARY FILE 12**

**Figure. Phenogroups characteristics.**

Three mutually exclusive phenogroups as determined by hierarchical clustering using the multiple correspondence analysis based on phenotypical information as input. The most distinct clinical and CMR characteristics are listed per phenogroup.

**
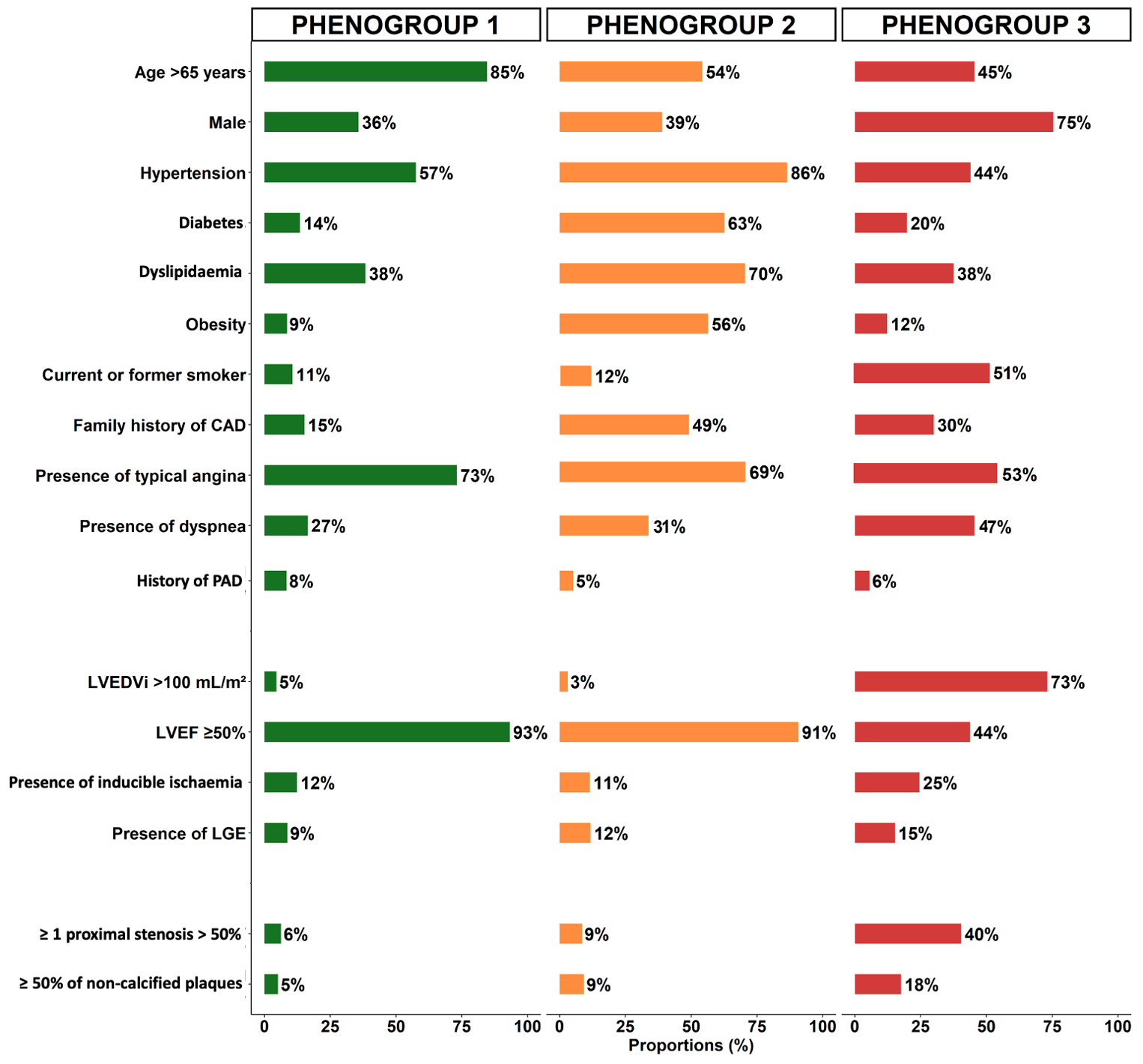
**

**SUPPLEMENTARY FILE 13**

**Figure. Box plots: Distribution of Cardiovascular Magnetic Resonance Parameters.**

Distribution of cardiovascular magnetic resonance (CMR) parameters: left ventricular end-diastolic volume indexed, LVEDVi (A) and left ventricular ejection fraction, LVEF (B).

These distributions suggested the difference between phenogroup 3 and phenogroups 1 and 2 regarding the value of LVEDVi and LVEF (for both p<0.001). Box plots show median (horizontal line) and interquartile range (extremities of the box).


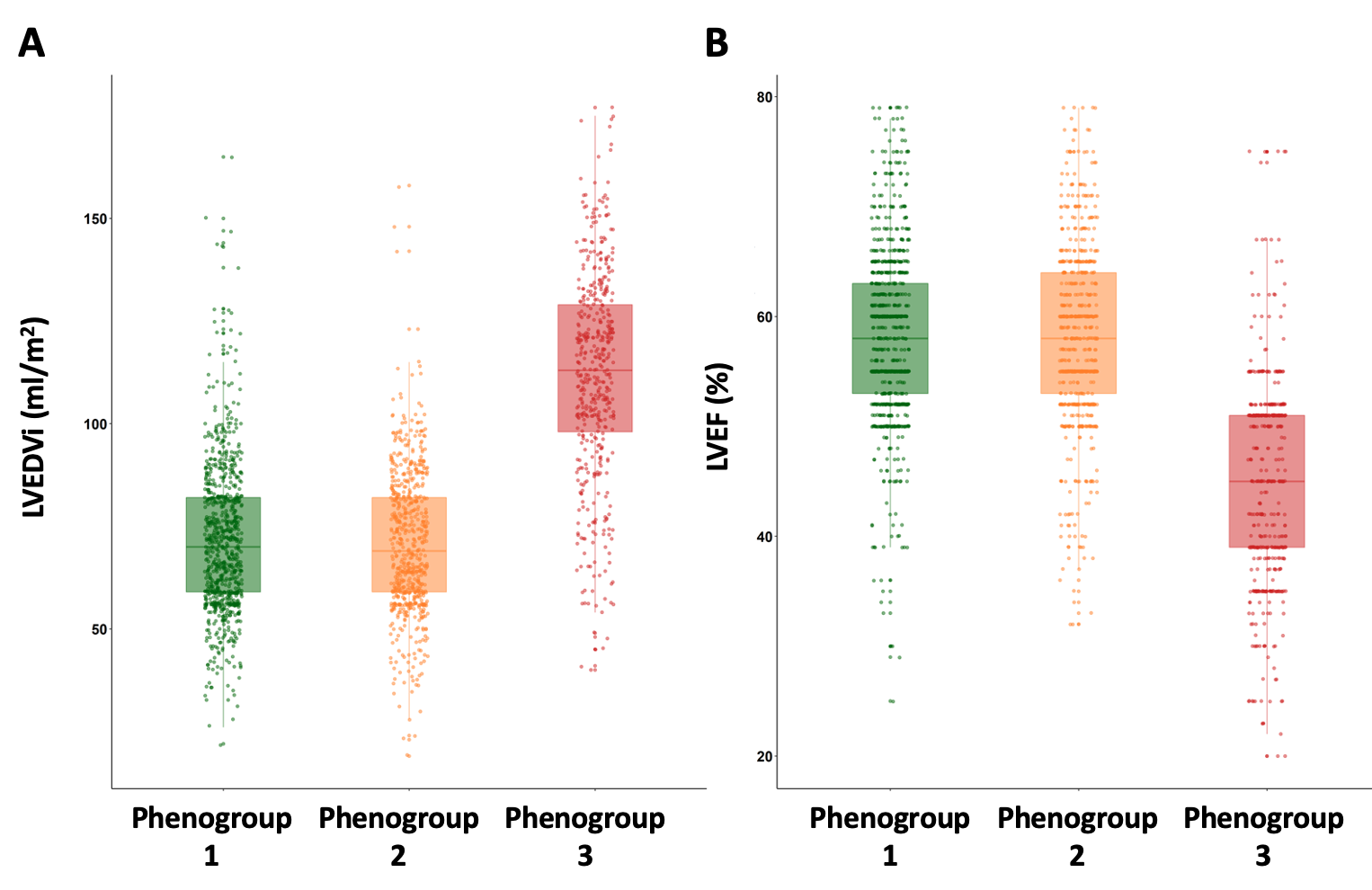


**SUPPLEMENTARY FILE 14**

**Table. Association between the proportion of non-calcified plaques and the presence of LGE with ischemic pattern within each phenogroup.**

|  | **Phenogroup 1**  **(N=854)** | **p-value** |  | **Phenogroup 2**  **(N=681)** | **p-value** |  | **Phenogroup 3**  **(N=480)** | **p-value** |  |
| --- | --- | --- | --- | --- | --- | --- | --- | --- | --- |
|  |  |  |  |  |  |  |  |  |  |
| **Unadjusted OR (95% CI)^*^** |  |  |  |  |  |  |  |  |  |
| Proportion of non-calcified plaques > 50% | 1.09 (0.80-2.22) | 0.67 |  | 1.16 (0.89-2.89) | 0.31 |  | 1.98 (1.09-3.29) | **0.041** |  |
|  |  |  |  |  |  |  |  |  |  |

^*^Unadjusted OR measures the association between the proportion of noncalcified plaques >50% by CCTA and the presence of LGE with ischemic pattern by CMR at baseline.

*Abbreviations: CI: confidence interval; LGE: late gadolinium enhancement; OR: odd ratio.*

**SUPPLEMENTARY FILE 15**

**Table. Sensitivity analysis: Association of phenogroups with adverse outcomes on Cox proportional hazard analysis.**

|  | **Phenogroup 1**  **(N=854)** |  | **Phenogroup 2**  **(N=681)** | **p-value^*^** |  | **Phenogroup 3**  **(N=480)** | **p-value**^†^ |  |
| --- | --- | --- | --- | --- | --- | --- | --- | --- |
|  |  |  |  |  |  |  |  |  |
| **Adjusted HR (95% CI)**^‡^ |  |  |  |  |  |  |  |  |
| MACE | 1.0 |  | 2.01 (1.30-3.00) | **<0.001** |  | 2.17 (1.38-3.35) | **<0.001** |  |
| CV mortality | 1.0 |  | 2.28 (1.32-3.97) | **<0.001** |  | 2.34 (1.28-4.39) | **<0.001** |  |
| All-cause mortality | 1.0 |  | 2.12 (1.30-3.47) | **<0.001** |  | 2.21 (1.28-3.85) | **0.003** |  |
|  |  |  |  |  |  |  |  |  |

^*^ The comparisons between PG1 and PG2 that were statistically significant with p<0.05 are shown in bold type.

^†^ The comparisons between PG1 and PG3 that were statistically significant with p<0.05 are shown in bold type.

^‡^ Covariates in the **model** by stepwise variable selection with entry and exit criteria set at the p≤0.1 level: ESC risk score (European score based on a modified SCORE project (https://www.escardio.org/Education/Practice-Tools/CVD-prevention-toolbox/SCORE-Risk-Charts), LVEF per 10%, presence of LGE, presence of ischemia, presence of ≥ 1 proximal stenosis > 50% and proportion of non-calcified plaques > 50%.

*Abbreviations: CI: confidence interval; CV: cardiovascular; HR: hazard ratio; MACE: major adverse cardiac events; PG: phenogroup.*

**SUPPLEMENTARY FILE 16**

**Table. Distribution of CCTA findings according to the occurrence of MACE in patients with ischemia.**

| **CCTA parameters** | **Ischemia**  **Without MACE**  **(N=197)** |  | **Ischemia**  **With MACE**  **(N=105)** | **p-value** |  |
| --- | --- | --- | --- | --- | --- |
|  |  |  |  |  |  |
| No. of proximal segments with stenosis >70% | 0.1 ± 0.4 |  | 0.5 ± 0.2 | **<0.001** |  |
| No. of segments with noncalcified plaques | 0.8 ± 0.8 |  | 1.6 ± 0.6 | **<0.001** |  |
|  |  |  |  |  |  |

**SUPPLEMENTARY FILE 17**

**Table. Association of the number of proximal segments with noncalcified plaques** **and the occurrence of MACE.**

|  | **Phenogroup 1**  **(N=854)** | **p-value** |  | **Phenogroup 2**  **(N=681)** | **p-value** |  | **Phenogroup 3**  **(N=480)** | **p-value** |  |
| --- | --- | --- | --- | --- | --- | --- | --- | --- | --- |
|  |  |  |  |  |  |  |  |  |  |
| **Unadjusted HR (95% CI)^*^** |  |  |  |  |  |  |  |  |  |
| Number of proximal segments with  noncalcified plaques | 1.57 (1.31-1.89) | **<0.001** |  | 1.73 (1.22-2.45) | **<0.001** |  | 2.06 (1.63-2.53) | **<0.001** |  |
|  |  |  |  |  |  |  |  |  |  |

^*^Unadjusted OR measures the association between the number of proximal segments with noncalcified plaques by CCTA and the occurrence of MACE.

*Abbreviations: CI: confidence interval; CV: cardiovascular; HR: hazard ratio; MACE: major adverse cardiac events.*
